# Loeffler on Diphtheria

**Published:** 1891-01-10

**Authors:** 


### THE PRACTITIONER'S MIRROR AND RETROSPECT.

*[The Editor will be glad to receive offers of co-operation and contributions from members of the profession. There is no doubt that much valuable material full of interest and instruction is at present lost to science. This is largely so in the case of (1) the younger and less-known members of the hospital staff (2) those connected with cottage hospitals, and (3) the great body of medical practitioners not attached to any institution. The co-operation of all is cordially invited to enable the Editor to make* The Hospital *of the utmost possible use and value to the profession. Specialists willing to review books on their special subjects are invited to communicate this fact at once. All letters should be addressed to* The Editor, The Lodge, Porchester Square, London, W.]

### MEDICINE.

### LOEFFLER ON DIPHTHERIA.

Prof. Löeffler, to whom is due the credit of identifying the specific and pathogenic bacillus of diphtheria, recommends a quarantine of not less than four weeks. The bacilli are present in the throat so long as the least trace of false membrane remains, and even for a few days longer. In dried fragments of membrane they retain their vitality for four or five months, a fact which demonstrates the absolute necessity for the thorough cleaning and disinfection of every article of furniture, and of the floor and walls of an infected room. How long they can live in the moist state he does not know, but thinks that they may do so for much longer periods, as out of the body they thrive and multiply at a temperature of 20 deg. C. [68 deg. F.], or under, and milk affords an excellent medium for their growth. We believe that the bacilli, as well as those of enteric fever and cholera, erysipelas, septicæmia, and tetanus exist in nature more or less universally, and perpetuate themselves independently of the presence of man. All morbid conditions of the pharynx are favourable to infection, and persons liable to sore or weak throats should be especially cautious, using antiseptic gargles frequently, when diphtheria or any form of suspicious sore throat prevails in a locality. Löeffler doubts the identity of the so-called diphtheritic affections of domestic animals with the disease in man, or their intercommunicability, and considers that Klein's observations on cats require further investigation.

